# Rethinking task failure in resistance training research: a framework for submaximal exercise prescription

**DOI:** 10.1007/s00421-026-06129-5

**Published:** 2026-01-24

**Authors:** William B. Hammert, Enrique N. Moreno, Robert W. Sallberg, Yujiro Yamada, Emily E. Metcalf, Evan W. Young, Ryo Kataoka, Jeremy P. Loenneke

**Affiliations:** 1 Department of Health, Exercise Science, and Recreation Management, Kevser Ermin Applied Physiology Laboratory, The University of Mississippi, P.O. Box 1848, University, MS 38677 USA; 2 School of Kinesiology and Nutrition. Applied Physiology Laboratory, The University of Southern Mississippi, Hattiesburg, MS USA

**Keywords:** Submaximal resistance training, Task failure, Skeletal muscle growth, Exercise prescription, Methodological sensitivity, Experimental design

## Abstract

Resistance exercise performed to task failure is often prescribed in muscle growth research to standardize effort and minimize between-participant variability. While effective for eliciting robust hypertrophic responses, this approach may also obscure meaningful differences attributable to manipulation of specific training variables, thereby reducing experimental sensitivity. The present perspective outlines a conceptual framework for implementing submaximal resistance exercise prescriptions to enhance methodological precision when investigating skeletal muscle growth. We discuss the potential limitations of task failure designs, emphasizing how maximal effort protocols can saturate muscle growth responses and constrain interpretability. We then provide a few considerations for applying submaximal models, including standardized pre-testing to determine individual maximum repetition capacities and strategies for exercise prescription and progression that maintain a submaximal intent. Important design factors include the selection of proximity to task failure, the choice between absolute (fixed offset from failure) and relative (percentage of maximal repetitions) frameworks, and the development of progression strategies. It is our opinion that anchoring training prescriptions to empirically derived pre-testing data will improve reproducibility, preserve between-group differences, and enhance the interpretability of muscle growth outcomes. Submaximal exercise prescriptions may thus offer a methodological foundation for advancing experimental design in resistance training research and refining our understanding of how specific training variables influence skeletal muscle growth adaptations.

## Introduction

Regular engagement in resistance exercise has been shown to be an effective strategy for increasing skeletal muscle size (Currier et al. 2023). As a result, a considerable amount of research has aimed to better understand the effects that manipulating different resistance training variables [e.g., load (Schoenfeld et al. 2017a; Lopez et al. 2021), set volume (Schoenfeld et al. 2017b; Hammert et al. 2023a), repetition duration i.e., “tempo”(Wilk et al. 2021), rest intervals (Grgic et al. 2017)] has on skeletal muscle growth adaptations. Many authors (Phillips and Winett 2010; Morton et al. 2015, 2019a; Fisher et al. 2018; Weakley et al. 2023) contend that deliberately manipulating these variables is of much less importance for stimulating growth than exercising with a high degree of voluntary effort (i.e., training to, or near task failure^1^). Indeed, we have previously suggested that including variation (Hammert et al. 2021) and progression (Hammert et al. 2024a) in a resistance training program geared towards maximizing one specific adaptation (e.g., skeletal muscle growth) is likely not necessary over short time frames (e.g., 6–12 weeks). Our group has also encouraged researchers to administer all sets to task failure rather than prescribing an arbitrary number of repetitions (e.g., 3 sets of 10 repetitions at 70% one-repetition maximum [1RM]) on the basis that doing so accounts for different levels of local muscular endurance (i.e., maximal repetitions that can be completed at a given load), and better ensures that all individuals receive a more homogenous stimulus for skeletal muscle growth (Dankel et al. 2017a). As with any methodological standard, however, it is important to remain open to emerging data and reevaluate the evidence from which such recommendations have been made. The purpose of the present article is to provide an updated perspective on exercising to task failure in muscle growth-focused resistance training research, with particular emphasis on whether such an approach truly allows for the most appropriate comparisons between protocols. More specifically, we identify some potential limitations of prescribing exercise to task failure for isolating the effects of individual training variables and propose the use of submaximal resistance exercise protocols as a methodological alternative to improve experimental sensitivity (i.e., ability to detect between-group differences) and facilitate continued advancement in resistance training studies designed to examine the role that manipulating different training variables may, or may not, have on skeletal muscle growth adaptations.

### Why prescribe exercise to task failure?

A major challenge in research comparing resistance training variables is the ability to isolate the impact of a specific training variable on muscular adaptations, as modifying one variable (e.g., exercise load) inevitably alters another (e.g., maximum number of repetitions) (Wernbom et al. 2006; Polito 2019). Researchers have traditionally tried to overcome this limitation by matching the exercise volume performed in one protocol to that of the other protocol(s) (Bemben et al. 2000; Holm et al. 2008; Burd et al. 2010). Exercise volume can be quantified in several ways, such as total training volume or “volume load” (i.e., total kg lifted; repetitions × load), relative volume load (i.e., sets × repetitions × %1RM), and/or the total number of weekly sets performed to or near task failure for a given muscle/muscle group (Schoenfeld et al. 2019; Nunes et al. 2021; Baz-Valle et al. 2022; Hammert et al. 2023a, 2024a). It should be acknowledged, however, that these metrics only provide a numerical estimate of the external demand imposed on the targeted muscle (i.e., the total amount of ‘work’ performed) (Hammert et al. 2024a). Training-induced skeletal muscle growth is ultimately a local biological process driven by mechanotransduction within the muscle itself (Marcotte et al. 2015; Lim et al. 2022). Thus, irrespective of how it is quantified, exercise volume will likely not represent the actual dose of loading experienced by the muscle fibers, as evidenced by it not having a fixed relationship with skeletal muscle growth (Morton et al. 2016; Hammert et al. 2024a). An additional aspect that should be considered is that equating volume using fixed repetition or set schemes can unintentionally constrain proximity to task failure across conditions. For example, if researchers equate total repetitions or volume load (e.g., 10 sets of 10 repetitions versus 5 sets of 10 repetitions), differences in repetition capacity across the loading spectrum may result in some participants not reaching a comparable proximity to failure, even when load adjustments are made. Given the aforementioned limitations, some researchers have suggested that the most practical way to control for differences in local muscular endurance and ensure a consistent growth stimulus across a study sample is to prescribe all sets to task failure (Dankel et al. 2017a). More specifically, it has been proposed that, because the former two methods of volume quantification are highly dependent upon the absolute load lifted and will differ drastically based on the total number of repetitions performed, reporting both the relative load lifted and number of sets performed (to task failure) would allow for better replication of data (i.e., improve the reproducibility of findings) and more appropriate comparisons across the literature (Dankel et al. 2017a). The methodology of prescribing all sets to task failure has also been shown to reduce the magnitude of variability in short-term responses (e.g., pre- to post-exercise changes in muscle thickness) compared to a fixed number of repetitions (Exner et al. 2023), which, for researchers, can be advantageous for yielding more precise point estimates, increasing statistical power, and ultimately reducing the sample size required to detect a given effect size.

The physiological rationale for prescribing all sets to task failure stems from Henneman’s size principle (1957), which describes the orderly recruitment of motor units from smallest to largest as contractile force demands increase (Denny-Brown and Pennybacker 1938). During sustained contractions, as lower-threshold motor units fatigue, higher-threshold units are progressively recruited to maintain force output (Fallentin et al. 1993). Exercising to the point where the muscle can no longer generate the force needed to complete another repetition would thus, in theory, exhaust and activate the full spectrum of motor units capable of being voluntarily recruited. If achieving high levels of muscle activation is indeed a key driver of skeletal muscle growth (Marcotte et al. 2015; Morton et al. 2015; Dankel et al. 2017a, b) then, assuming a sufficient amount of contractions are being performed per set/duration of time, training to task failure should provide a similar growth-inducing stimulus regardless of the training protocol employed. In support of this contention, Morton et al. (2019b) reported comparable glycogen depletion (indicator of muscle activation) in both type I and type II fibers following low-load (30% 1RM) and high-load (80% 1RM) knee extension exercise performed to task failure. Modifying repetition duration within each loading condition further altered the number of repetitions completed, but did not differently affect muscle fiber activation or anabolic signaling (Morton et al. 2019b). These findings, together with a broader body of literature reporting similar hypertrophic adaptations after periods of high- and low-load resistance training performed to task failure (Schoenfeld et al. 2017a), support the notion that exercising to task failure can provide a similar stimulus for skeletal muscle growth across diverse training approaches.

### Potential limitations of prescribing exercise to task failure?

While prescribing exercise to task failure may help standardize the hypertrophic stimulus across participants (Dankel et al. 2017a; Exner et al. 2023), it also carries methodological drawbacks that may complicate efforts to isolate the effects of individual training variables, particularly in studies aiming to tease out the unique contributions of different programming variables. It is entirely plausible, for example, that if two training protocols are both sufficient to fully recruit the available motor unit pool, each will be similarly effective in terms of stimulating skeletal muscle growth. Numerous studies have compared training protocols that differ in one specific variable [e.g., load (Schoenfeld et al. 2017a; Lopez et al. 2021), repetition tempo (Wilk et al. 2021), rest intervals (Grgic et al. 2017)] and reported comparable increases in skeletal muscle size when all sets were performed to task failure. Similar patterns of results have been observed in the blood flow restriction literature, despite differences in total exercise volume (Fahs et al. 2015; Farup et al. 2015; Pignanelli et al. 2020), as well as studies that have investigated more “advanced” training techniques, such as pre-exhaustion (Trindade et al. 2019) and drop set training (Ozaki et al. 2018; Coleman et al. 2022). From a broader perspective, the potential issue of prescribing exercise to task failure in research settings mirrors a recent point-counterpoint discussion regarding rodent models of overload. Burke et al. (2024) argued that synergistic ablation remains powerful for uncovering hypertrophy mechanisms because the chronic mechanical overload it produces elicits a growth response so remarkable that relevant molecular and cellular events are more easily detectable above biological noise. However, as the counterpoint authors, Roberts et al. (2024) noted, this “sledgehammer” stimulus can also obscure whether the observed mechanisms are universally required for hypertrophy across other, less extreme overload models. We contend that a similar analogy can be made to prescribing resistance exercise to task failure in human research studies. That is to say, the very robustness of the stimulus may mask potential differences attributable to manipulation of individual training variables, ultimately limiting the sensitivity of such designs to detect between-group differences in muscle growth outcomes.

An additional, practical limitation of prescribing training to task failure is that it may not reflect how most people perform resistance exercise outside the laboratory. Observational and experimental data indicate that individuals train closer to failure when supervised than when training alone; for example, Steele et al. (2025) reported higher rating of perceived exertions values with supervision in both real-world and experimental settings, suggesting that unsupervised individuals typically self-select submaximal effort. This may be particularly relevant given that resistance training is often prescribed using perceptual anchors (e.g., ratings of perceived exertion or repetitions in reserve (Zourdos et al. 2016; Helms et al. 2016), yet it is not uncommon for individuals to misjudge their true proximity to task failure when relying on these subjective scales. Thus, task failure designs not only reduce methodological sensitivity, but also lack ecological validity for understanding how training variables influence adaptation in settings where individuals do not routinely (or willingly) train to task failure. From an application standpoint, these data highlight the importance of using submaximal models, which may better reflect real-world resistance training behaviors and help identify which training variables influence adaptation under more typical, self-selected effort conditions.

### If not task failure, then what?

In a mechanistic study, Smith et al. (Smith et al. 2012) examined the muscle protein synthetic response to feeding in older adults before and after 3 months of exercise training. To avoid a “ceiling effect”, the authors (Smith et al. 2012) intentionally provided a meal containing ~ 10–15 g of protein, an amount they hypothesized would stimulate protein synthesis without maximizing the response. We wish to suggest that a similar logic can be applied to resistance training research. To illustrate, rather than prescribing sets to task failure, researchers might consider using submaximal protocols that are individualized to each participant’s local muscular endurance capabilities and avoid reaching task failure. In doing so, between-group/condition differences in the applied stimulus are more likely to be preserved, thereby providing a more sensitive experimental platform (than training to task failure) and allowing for a more appropriate determination of the influence of, or lack thereof, specific training variables on training-induced adaptations.

Our group has recently conducted a series of experiments using submaximal resistance exercise prescriptions. More specifically, we have applied this framework in both acute (Hammert et al. 2024b, 2025; Yamada et al. 2025a, b) and chronic (Kataoka et al. 2025) resistance training studies, with the goal of isolating the independent effects of blood flow restriction and comparing different methods of pressure application. Although still in its infancy, we believe this approach provides a starting point for improving the sensitivity of studies examining the influence of specific training variables on skeletal muscle growth. To facilitate the use of these methods in future studies, we have outlined some considerations below.

### General considerations for pre-testing

The fundamental principle of submaximal designs is that prescribed exercise should be lower than each individual’s maximum repetition capacity (i.e., local muscular endurance capabilities) to avoid any potential ceiling effect associated with reaching task failure. Just like any measurement, it is essential for researchers to use standardized testing procedures when assessing maximum repetition capacities under the to-be-investigated protocol. Beyond standardizing exercise form (e.g., completing an appropriate range of motion), some aspects that we believe are especially important to consider during pre-testing are the repetition duration (tempo) and number of exercise sets. Repetition tempo should be controlled for during testing (e.g., paced by a metronome) and kept constant during training, as it has been shown that altering the concentric and eccentric muscle action velocities can influence the total number of repetitions to task failure across a wide range of exercise loads (i.e., 30–80% 1RM) (Sakamoto and Sinclair 2006; Morton et al. 2019b). Failure to standardize cadence during pre-testing and training risks introducing confounding if participants subsequently exercise using faster or slower repetitions, since any observed differences in training outcomes might then reflect differences in time under tension rather than the training variable of interest. In addition to repetition tempo, the number of sets performed during pre-testing should mirror the number prescribed during the intervention itself. For example, if participants are expected to train with two sets during the intervention, then maximum repetition testing should also involve two sets, so that exercise prescription reflects both the individual’s initial capacity and the performance decrement that occurs with repeated sets to task failure (Kataoka et al. 2025). It should be acknowledged, however, that higher volume training protocols (i.e., number of sets) will likely saturate the growth-stimulus (Hammert et al. 2023a; Moreno et al. 2024) and thus reduce the ability to detect between-group differences. In other words, once a certain set-volume threshold is reached, additional sets, even if submaximal, may produce diminishing returns and make it more difficult to observe any effects of the manipulated training variable. Of course, if researchers are specifically interested in identifying the set-volume at which a saturated growth response emerges during submaximal training, then including conditions with varying numbers of sets would be appropriate. Researchers may also consider incorporating a test–retest approach, whereby maximum repetition capacity is assessed on two separate days and the values are averaged to provide a more stable estimate of each participant’s repetition capacity. While this increases both research and participant burden, such an approach may help account for day-to-day variability in performance, particularly among untrained individuals. We acknowledge that repeated testing may introduce practice (i.e., familiarization) effects and transient muscle soreness; however, because participants are to be randomized after pre-testing, any such effects would be expected to be evenly distributed across groups/conditions, thereby minimizing the potential for systematic bias.

Our laboratory has attempted to balance the aforementioned considerations by working primarily with a two-set model. In our acute work (Hammert et al. 2024b, 2025; Yamada et al. 2025a, b), we have used the first set to quantify each individual’s maximal repetition capacity and the second to measure performance decline, using these data to prescribe exercise for subsequent visits. More recently, we applied and refined these methods in a 6-week blood flow restriction training intervention (Kataoka et al. 2025). During pre-testing, participants completed three total sets of exercise. First, they performed a single set to failure to determine their maximum capacity under blood flow restriction, then rested 5-minutes, and subsequently completed two additional sets of exercise. The initial set was performed at 70% of their maximum repetition capacity to replicate the stimulus used in training, followed by a second set to task failure to determine the number of repetitions to be prescribed for the second set during training [see to the Methods section of Kataoka et al. (2025) for more detail]. Such methodologies allowed us to keep participants from reaching task failure for the entirety of the training intervention and detect differences between training groups, demonstrating feasibility and experimental sensitivity. Taken together, a two-set model approach seemingly provides a practical and methodologically rigorous framework for prescribing submaximal exercise while preserving between-group differences and avoiding a saturated stimulus for skeletal muscle growth. Furthermore, the inclusion of a failure-based training group as a “positive control” can provide additional strength to submaximal designs, as it enables researchers to evaluate whether between-group differences emerge regardless of proximity to failure.

### Specific considerations for pre-testing

While the previous paragraphs highlight a few considerations that provide a foundation for study designs, the exact implementation of submaximal exercise prescriptions will ultimately be dictated by the research question. An important decision is whether the training protocols should differ only with respect to the manipulated training variable (e.g., load, repetition tempo, rest intervals), or whether they should also be equated for the level of effort put forth (i.e., the proximity to task failure).

If the goal is to test the effects of a given training variable, it follows that training be prescribed relative to each individual’s maximum repetition capacity using the “reference standard” training protocol. For example, in a hypothetical comparison between high- and low-load resistance training, researchers could have participants complete two sets of as many repetitions as possible using the heavier of the to-be-investigated loads and then prescribe exercise at a fixed proportion (e.g., percentage of maximum repetitions) of this capacity for both training groups. Experimentally, the high- and low-load protocols would differ primarily with respect to the magnitude of load lifted per repetition. Yet, it should be acknowledged that, because individuals can complete more repetitions (to task failure) using lower loads (Morton et al. 2019b; Nuzzo et al. 2023), the proximity to task failure, and thus relative effort required to complete each set, would inevitably differ between groups. As such, the observed adaptations (or lack thereof) would reflect the isolated influence of load under conditions of unequal effort. When designing a study to isolate the effects of a given training variable, it is therefore important to consider which specific training protocol should be considered the reference standard for exercise prescription. For example, in the abovementioned hypothetical comparing high- and low-load resistance training, electing to use the lower exercise load as the reference standard training protocol can become problematic because it requires prescribing training under the assumption that participants could consistently perform the same number of repetitions achievable with lower loads using heavier loads [(i.e., a scenario that is unlikely given that heavier loads inherently limit repetitions to task failure (Buckner et al. 2020))]. Likewise, when investigating different repetition tempos, using the faster paced training protocol as the reference standard may pose issues for prescribing training, as researchers would be assuming that participants could consistently perform the same number of repetitions using slower repetition tempos, which may not always hold true (Morton et al. 2019b). As such, for those reasons, it is our position that whichever condition allows for fewer repetitions to task failure will serve as the better reference, namely because it would provide a more conservative and reproducible anchor for prescribing submaximal training, thereby reducing the likelihood that participants unintentionally exercise to task failure. Indeed, the last thing researchers would want when employing a submaximal design is for participants to inadvertently exercise to task failure (Hammert et al. 2023b), as this would negate the very purpose of using a submaximal approach in the first place.

In comparison to variable-isolated (i.e., non-effort matched) designs, if the goal is to compare high- versus low-load resistance training under conditions of matched relative effort, it makes conceptual sense that training be prescribed based on each individual’s maximum repetitions achievable under the specific loading condition. In this scenario, participants would complete two sets to task failure using both the heavier and lighter loads during pre-testing and exercise would be prescribed at a fixed proportion of each respective condition’s maximum capacity. The proximity to task failure and thus relative effort is now more accurately standardized between the training groups, thereby enabling researchers to investigate the effects of exercise load following effort-matched high- and low-load resistance training. We, and others, have used similar methods for exercise prescription purposes. For example, our initial submaximal design prescribed exercise at 70% of each individual’s maximum repetition capacity achieved during pre-testing with and without blood flow restriction (Hammert et al. 2024b). Likewise, Keramidas et al. (2012) used incremental exercise testing with and without blood flow restriction to set exercise intensities for a 6-week interval training study, ensuring that participants exercised at the same relative intensity under each condition. In another study (Mendonca et al. 2011) examining the acute effects of cigarette smoking on cardiac autonomic function during exercise, the authors had participants complete graded exercise testing under smoking and control conditions to identify workloads corresponding to the same fractional utilization of VO_2peak_, and thus allow subsequent experimental visits to be matched for relative intensity.

From a research perspective, both variable-isolated and effort-matched submaximal prescriptions can be advantageous in helping avoid the potential sledgehammer effect of prescribing exercise to task failure. Whether the former (i.e., variable-isolated), the latter (i.e., effort-matched), or a combination of the two is employed in study designs should be chosen based on the question of interest. In general, the former ensures that, at least on paper, the protocols differ only in the manipulated variable, which may be a necessary first step for isolating its independent effect on skeletal muscle growth outcomes. Subsequent work may then test whether differences (or lack thereof) between different training protocols persist when equating for relative effort, helping determine the extent to which effort itself contributes to skeletal muscle growth adaptations. For example, if two submaximal protocols are matched for relative effort yet produce different muscle growth, it would suggest that the manipulated training variable exerts an independent effect. If similar muscle growth is observed under effort-matched conditions, however, it would indicate that the manipulated variable contributes little beyond the influence of relative effort itself. Lastly, researchers can include both variable-isolated and effort-matched protocols to examine how both the manipulated training variable and relative effort influences skeletal muscle growth outcomes.

### Considerations for prescribing training

Having established some important pre-testing considerations, a logical next step is to discuss how such information can help guide the prescription of submaximal training protocols. In this context, we have chosen three important considerations to highlight; namely, how far from task failure exercise should be prescribed, whether an absolute (e.g., fixed number of repetitions away from failure) or relative (e.g., percentage of maximum repetitions) framework should be used, and how, if at all, the training will be progressed throughout the intervention.

### How far away from task failure?

In terms of how far from task failure participants should train, it is important to acknowledge that there is likely a point at which a set becomes “close enough” to task failure that it does not provide a physiologically distinct stimulus for skeletal muscle growth than would be expected by training to task failure (Fisher et al. 2022). Exactly where that lies remains speculative. It has been demonstrated that terminating sets with perceived proximity of 1 to 2 repetitions in reserve produced similar muscle growth as training to task failure (Refalo et al. 2024). However, the fact that other aspects of the Refalo et al. (2024) study design were manipulated (e.g., previous training volume, diet) complicates definitive conclusions regarding the role of proximity to failure in promoting muscle growth. Moreover, it is possible that the use of a high training volume protocol may have saturated the hypertrophic stimulus (i.e., potentially masked between-condition differences). In another study by Hermann et al. (2025), the authors compared a group that performed a single set to task failure per exercise, with verbal encouragement from the researchers, to one that had participants exercise, without verbal encouragement, and subjectively terminating each exercise set when they believed they were 2 repetitions away from task failure. While conceptually distinct, it seems reasonable to suggest that both groups were effectively training at or near maximal effort, which if true, may explain why little evidence was found to support greater muscle growth for the group that trained to task failure with verbal encouragement (Hermann et al. 2025). Taken together, these findings suggest that the ability to detect differences between training at 1–2 perceived repetitions shy of task failure and task failure is likely minimal, and that submaximal designs should place a greater emphasis on prescribing larger proximities away from task failure. Perhaps more importantly, even when researchers prescribe submaximal exercise away using the repetition in reserve approach, it is impossible to know with certainty how close participants actually are from task failure, thus underscoring a fundamental limitation of subjective measures of proximity to failure.

### Absolute versus relative prescription frameworks

Whether exercise should be prescribed using an absolute (e.g., fixed number of repetitions away from failure) or relative (e.g., percentage of maximum repetitions) framework is another important methodological consideration. Assuming that maximal effort is given at pre-testing, it follows that either framework would offer a more objective alternative for prescribing submaximal training compared to perceived repetitions in reserve.

Our laboratory has previously employed the relative framework approach, prescribing exercise at 70% of each participant’s maximum repetition capacity. In doing so, we ensured consistency within individuals across repeated sessions in both acute studies (Hammert et al. 2024b, 2025; Yamada et al. 2025a, b) and chronic (Kataoka et al. 2025) interventions, as demonstrated by all participants completing the prescribed repetitions in each set. On the one hand, using the relative framework offers an advantage because it scales exercise volume in proportion to each participant’s endurance capacity, providing a link between pre-testing and exercise prescription (i.e., for training). On the other hand, it becomes limited by the possibility that a fixed percentage of repetitions may not represent an equivalent distance from task failure between conditions. For example, prescribing the same percentage of maximum repetitions under low-load resistance exercise with blood flow restriction and the same low-load exercise without blood flow restriction will likely correspond to different proximities to task failure (i.e., absolute number of repetitions from task failure) (Hammert et al. 2024b), potentially leading to different physiological stimuli. Importantly, however, such differences in effort may not necessarily be confounding if they represent an innate feature of the intervention (e.g., if exercising under blood flow restriction inherently induces greater effort). Yet, from a methodological standpoint, the primary concern is whether these effort-related differences align with the specific research aim; i.e., whether the goal is to isolate the effect of the intervention itself or to equate effort across conditions to examine the contribution of a given variable.

Based on the previously mentioned points, some researchers may prefer to prescribe exercise based on an absolute (i.e., fixed) number of repetitions away from task failure. The absolute framework draws from the same conceptual logic as the repetition in reserve approach, but anchors prescriptions directly to each participant’s empirically measured maximum capacity obtained during pre-testing. By defining a fixed distance from task failure (e.g., 5 repetitions short of the measured maximum), researchers can prescribe submaximal training in a reproducible manner, while minimizing reliance on subjective self-estimation. To illustrate, if an individual’s maximum repetition capacity during a single set of exercise with blood flow restriction was 30 repetitions, then they would be prescribed 25 repetitions (i.e., 5 repetitions below their measured maximum with blood flow restriction). If that same individual was able to complete 40 repetitions without blood flow restriction, then the analogous prescription would be 35 repetitions (i.e., 5 repetitions below their measured maximum without blood flow restriction). It should be noted, however, that the absolute framework introduces its own scaling problems. That is, removing a fixed number of repetitions (e.g., 3 repetitions) may not produce an equivalent reduction in effort across different training paradigms. For instance, subtracting 3 repetitions from a high-load set with a maximum of 10 repetitions reduces effort by 30%, whereas the same adjustment in a low-load set with a capacity of 30 repetitions reduces effort by only 10%. While effort is not a linear function of the percentage of repetitions completed (Steele et al. 2017), and likely is influenced by factors such as load-specific motor unit recruitment patterns, contraction velocity changes (Morton et al. 2019b), and the nonlinear rise in fatigue as task failure approaches, any fixed subtraction will nonetheless shift effort to a different degree depending on the repetition range and loading condition. Thus, the absolute approach differs from the relative proportion framework in its dimension of standardization. More specifically, it prescribes exercise based on a defined number of repetitions away from task failure rather than proportional effort, which may still result in unequal relative intensites between training protocols.

Neither framework is without limitation(s), and both offer distinct trade-offs. The percentage-based (relative) approach will likely represent the more proportional and scalable method for prescribing submaximal exercise, as it maintains a constant ratio between pre-tested capacity and training prescription. Such proportionality, however, does not guarantee an equal physiological effect as it cannot fully equate relative effort between different training protocols. In contrast, the absolute framework anchors prescriptions to a fixed distance from task failure, promoting clearer standardization of effort within a condition (i.e., absolute number of repetitions from task failure). Accordingly, the choice between absolute and relative frameworks should depend on the specific research question; that is, whether the goal is to isolate the effect of a training variable (favoring an absolute framework) or to equate relative effort between conditions (favoring a relative framework). Future work directly comparing these frameworks may help clarify which strategy better preserves submaximal intent and experimental sensitivity.

### Progression in submaximal designs

While prescribing exercise below task failure provides a safeguard against ceiling effects, it also raises the question of whether the training stimulus should incrementally increase over time. From a design standpoint, given that exercise prescriptions are anchored to maximum repetition capacities established during pre-testing, progression must logically take the form of gradually increasing repetitions to maintain the intended submaximal framework. A potential concern in this respect is that moving participants too close to task failure may eventually converge on a stimulus indistinguishable from training to task failure, ultimately reducing the ability to detect between-group differences and negating the methodological advantages of a submaximal design.

One possible strategy is to begin with a conservative buffer away from task failure (e.g., 70% of maximum pre-testing repetitions) and then progressively increase the prescribed proportion across weeks, thereby moving participants closer to failure as adaptation occurs. Importantly, if progression is capped at a point deemed “close enough” to failure (e.g., 85–95% of maximum repetitions), then the training protocol would likely still provide a sufficient stimulus for muscle growth without introducing the confounding effects of training to task failure. As an example, our recent 6-week blood flow restriction training study (Kataoka et al. 2025) employed a progressive, submaximal model, beginning at 70% of maximum repetitions and increasing to 95% by the final week (i.e., 5% increase per week), which ensured that the training remained submaximal for the entirety of the intervention, and minimized the risk of saturating the stimulus. Researchers may also consider re-testing maximum repetition capacities at periodic intervals to maintain an adequate stimulus while still staying below failure. Any such changes in maximum repetition capacities may reflect adaptations in local muscular endurance, and failure to account for these changes could result in participants training progressively further from the intended proximity to failure as the intervention progresses. However, to avoid introducing bias, non-exercise control groups must also undergo re-testing to account for any systematic effect of repeated testing (Spitz et al. 2020). If maximum repetition capacities are not re-evaluated during the intervention, a post-study maximal repetition test may provide useful insight into how far participants were training from failure and whether the chosen progression strategy delivered a sufficiently challenging stimulus. In any case, the decision of whether and/or how to implement progression should balance the need to maintain a robust growth stimulus with the need to preserve methodological sensitivity to between-group differences.

## Conclusion

In recent years, it has become increasingly common for researchers to prescribe all sets of resistance exercise to task failure as a way to standardize the hypertrophic stimulus and minimize between-participant variability. However, the very robustness of this methodology may also obscure meaningful differences attributable to manipulation of specific training variables, thereby limiting experimental sensitivity. It is our position that submaximal exercise prescriptions may provide a more effective alternative for experimental designs seeking to isolate the effects of individual programming variables on skeletal muscle growth. Indeed, by anchoring training to each participant’s maximum repetition capacity, researchers can prescribe exercise below failure, thereby mitigating ceiling effects while maintaining a reproducible stimulus. Some important considerations for implementing submaximal designs in training studies include: (1) determining an appropriate proximity from task failure, recognizing that 1–2 repetitions away is unlikely to represent a physiologically distinct stimulus; (2) selecting between absolute versus relative frameworks; and (3) deciding whether, and how, to incorporate progression, balancing the need for an adequate training stimulus with the necessity of maintaining a submaximal intent. Beyond experimental control, these frameworks may also have practical relevance for exercise prescription, particularly in populations for whom training to task failure is impractical or undesirable, by providing a reproducible yet flexible alternative to failure-based programming. Be that as it may, future work is needed to further refine these approaches, for example, by investigating different proximities from task failure, testing the feasibility of absolute prescription frameworks as well as single- and multi-set protocols, and evaluating the role of progression and re-testing, to further advance the field and improve the interpretability of resistance training research studies. To facilitate translation of these concepts into experimental research, we have provided two complementary frameworks. Table 1 outlines standardized procedures for pre-testing and establishing maximal repetition capacities under submaximal resistance-training designs, whereas Table 2 extends these principles to training prescription and progression. Together, these frameworks provide a starting point illustrating how experimental protocols can be anchored to pre-testing data, maintain submaximal intent, and preserve methodological sensitivity across common resistance-training variables (e.g., exercise load, tempo, rest intervals).

**Table 1:** Framework for pre-testing in submaximal resistance-training designs, used to establish each participant’s maximal repetition capacity prior to training

Design Type	Purpose	Concept / Logic	Implementation	Experimental Considerations	Limitation(s)	Example
*Variable-Isolated*	To isolate the effect of the manipulated training variable while allowing relative effort to differ.	Uses a single “reference” protocol (typically the one with the lower maximum repetition capacity) to standardize testing and ensure conservative submaximal prescriptions.	Conduct set(s) to task failure using the reference protocol to quantify maximum repetition capacity and performance decline. Prescribe all training relative to this value.	Ideal for research seeking to determine whether the manipulated variable itself drives adaptation.	Cannot discern whether adaptations stem from effort differences or the intended variable.	Pre-test maximal repetitions at 80% 1RM (high-load) and use this value as the reference to prescribe both high- and low-load training (in this example 70% of high-load maximum capacity) • Participant performs 10 repetitions to task failure using 80% 1RM. • Training is prescribed as 7 repetitions at 80% 1RM (high-load group) or 7 repetitions at 30% 1RM (low-load group).
*Effort-Matched*	To equate proximity to task failure across all training groups, reducing confounding from effort differences.	Determines each condition’s own maximal repetition capacity so prescriptions can reflect equal proportional effort.	Conduct set(s) to task failure under each experimental condition to define maximum repetition capacity and performance decline. Prescribe subsequent training relative to each condition’s own capacity.	Suited for testing examining whether a given variable affects adaptation independent of effort.	Requires more pre-testing (multiple failure assessments).	Pre-test maximal repetitions at both 80% 1RM (high-load) and 30% 1RM (low-load), then prescribe 70% of each condition’s maximum capacity. • Participant performs 10 repetitions to task failure using 80% 1 RM and 30 repetitions using 30% 1RM. • Training is prescribed as 7 repetitions (high-load) or 21 repetitions (low-load).

The variable-isolated design standardizes exercise/training parameters except the manipulated training variable, allowing relative effort to differ between conditions, whereas the effort-matched design standardizes proximity to task failure across conditions to control for effort-related confounding. Both approaches require control of repetition tempo, range of motion, rest intervals, and exercise setup to ensure reproducibility and meaningful interpretation of pre-testing data

**Table 2:** Framework for training prescription and progression in submaximal resistance-training designs, expanding on the pre-testing approaches described in Table 1

Design Type	Prescription Framework	Purpose	Concept / Logic	Implementation	Experimental Considerations	Limitation(s)	Example
***Variable-Isolated***	Absolute	To maintain distinct stimuli between conditions while holding effort a fixed distance below task failure.	Anchors all conditions to the reference protocol’s pre-tested maximal repetition capacity, prescribing a constant number of repetitions short of task failure.	Prescribe each set a fixed distance from the reference capacity. Progress gradually, while keeping the same buffer away from task failure.	Simplifies comparisons across protocols; differences in effort are allowed to isolate the manipulated variable.	Absolute number from task failure may scale unevenly across higher versus lower repetition protocols, thus altering relative intensity.	Pre-test maximal repetitions at 80% 1RM (high-load) and use this value as the reference to prescribe both high- and low-load training. • Participant performs 10 repetitions to task failure using 80% 1 RM. • Training is prescribed as 5 repetitions below task failure (high-load group = 5 repetitions at 80% 1RM; low-load group = 5 repetitions at 30% 1RM). • Training is progressed by 1 repetition every 2-weeks (7 repetitions prescribed for both groups during week 6).
	Relative	To scale effort proportionally while still isolating the manipulated variable.	Prescribes exercise as a fixed percentage of the reference protocol’s maximum.	Prescribe all conditions at the same percentage of the reference maximum. Progress by raising the percentage incrementally.	Maintains proportional workload relative to initial capacity.	Relative percentages may yield unequal absolute distances from failure, introducing effort disparities.	Pre-test maximal repetitions at 80% 1RM (high-load) and use this value as the reference to prescribe both high- and low-load training. • Participant performs 10 repetitions to task failure using 80% 1 RM. • Training is prescribed as 70% of maximum capacity (high-load = 7 repetitions at 80% 1RM; low-load = 7 repetitions at 30% 1RM). • Training is progressed by 10% every 2-weeks (i.e., 9 repetitions prescribed for both groups during week 6).
***Effort-Matched***	Absolute	To equate distance from failure across conditions and reduce variability in perceived effort.	Anchors prescriptions to each condition’s own maximal capacity, prescribing a fixed number of repetitions below task failure.	Prescribe all sets a fixed number of repetitions short of each condition’s maximum. Can also periodically re-test to maintain a consistent buffer as adaptation occurs.	Offers clear interpretability of “distance from failure” but may not result in similar total work between conditions.	Different repetition ranges create unequal proportional effort. Frequent re-testing required may introduce bias if controls are not used.	Pre-test maximal repetitions at both 80% 1RM (high-load) and 30% 1RM (low-load). • Participant performs 10 repetitions to task failure using 80% 1 RM and 30 repetitions using 30% 1RM. • Training is prescribed as 5 repetitions below task failure (high-load group = 5 repetitions at 80% 1RM; low-load group = 25 repetitions at 30% 1RM). • If no retesting occurs, training is progressed by 1 repetition every 2-weeks (high-load group = 7 repetitions at 80% 1RM during week 6; low-load group = 27 repetitions at 30% 1RM during week 6). • If retesting occurs, training is progressed by prescribing 70% of each condition’s new maximum capacity. E.g., participant performs 15 repetitions to task failure using 80% 1 RM and 40 repetitions using 30% 1RM (high-load = 10 repetitions at 80% 1RM; low-load = 35 repetitions at 30% 1RM).
	Relative	To standardize proportional effort and match relative intensity between protocols.	Prescribes exercise as a fixed percentage of each condition’s maximal capacity.	Determine each condition’s capacity during pre-testing. Prescribe a submaximal percentage of the repetition capacity and progress conservatively.	Enhances internal validity by matching effort and minimizing confounding.	Equal proportional effort does not ensure similar physiological stimuli.	Pre-test maximal repetitions at both 80% 1RM (high-load) and 30% 1RM (low-load). • Participant performs 10 repetitions to task failure using 80% 1 RM and 30 repetitions using 30% 1RM. • Training is prescribed as 70% of each condition’s maximum capacity (high-load = 7 repetitions at 80% 1RM; low-load = 21 repetitions at 30% 1RM). • If no retesting occurs, training is progressed by 10% every 2-weeks (high-load = 9 repetitions at 80% 1RM during week 6; low-load = 27 repetitions at 30% 1RM during week 6). • If retesting occurs, training is progressed by prescribing 70% of each condition’s new maximum capacity. E.g., participant performs 15 repetitions to task failure using 80% 1 RM and 40 repetitions using 30% 1RM (high-load = 10 repetitions at 80% 1RM; low-load = 28 repetitions at 30% 1RM).

The absolute framework maintains a fixed distance from task failure (e.g., 3–5 repetitions), whereas the relative framework scales effort as a percentage of maximal repetition capacity (e.g., 70%). Either framework can be implemented within either variable-isolated or effort-matched designs, depending on whether the goal is to preserve differentiation between conditions or to equate relative effort across them. Prescriptions should remain anchored to pre-testing/re-testing data, employ gradual and controlled progression, and ensure that exercise remains submaximal throughout the intervention to preserve methodological sensitivity

## Abbreviations

1RM: One-repetition maximum

## Footnotes

1 To clarify language, in the present manuscript the term *task failure* is used to refer to the inability to complete an additional repetition while maintaining the prescribed task requirements (e.g., range of motion, cadence, and technique), thereby differentiating it from other definitions used throughout the literature, which may occur at different points depending on task constraints and participant interpretation. For a more comprehensive discussion of proximity to failure and related definitions in resistance training, see Steele et al. (2017) and Refalo et al. (2022).
